# A Tropical Poor House

**Published:** 1897-10-16

**Authors:** 


### A TROPICAL POOR HOUSE.

### THE ALMSHOUSE, GEORGETOWN, BRITISH GUIANA.

The Almshouse at Georgetown is an institution for the indigent poor of the colony somewhat similar to our own workhouses, but in many points it more resembles an enormous incurable hospital, there being no really able-bodied inmates, and all are adults. The orphan asylum provides for the young. A large number of the inmates are old, partially if not entirely helpless, many crippled and otherwise deformed. The building has an imposing appearance, and is entered by handsome gates. The court is laid out as a garden, gay with flowers. To the right is the old block, containing on the ground floor the superintendent's office, linen rooms, and other offices. A wide gallery runs along the whole block. The superintendent's quarters are spacious and airy. In the centre of the block is a large female ward with about fifty beds; then, strange anomaly, the male nurse's quarters; then two rooms for the matron. The matron's bed-room is dark, and not airy enough for the habitation of a European in a tropical country; the sitting-room is good but very public, as the inmates must pass both door and window to enter the ward, and this publicity is added to by the fact that the staircase appears a favourite lounge; and the place is permeated by unpleasant odours. In the gallery attached to this block when I visited it a group of twenty to thirty women were squatting eating from three large tin dishes, fishing out bits with their fingers, and wiping their hands on their clothes.

The women's infirmary ward was not sweet, and the chamber utensils and other articles were very dirty; the beds and blankets were all fairly good, but the place had an air lacking supervision and orderliness. The superintendent complained of the great difficulties that existed, one being owing to the inmates insisting on hiding food, &c., in the beds. This happened often with the nurse's connivance, and the superintendent appeared powerless to prevent it. Nor is this surprising, as only one nurse and an assistant were provided to attend to 240 inmates during both day and night. The matron was supposed to act not only as head nurse, but sewing mistress.

The largest ward had an harmonium, and occasional services were held and singing was permitted. Nurses are provided with brown uniform. The male block is better in construction, well built and ventilated, and in every respect better arranged, cleaner, and in much better order. The male nurses are clean and smart. The superintendent, Mr. Cook, is most anxious to improve matters, and has done a good deal, and will doubtless in time persuade the authorities to make other much-needed improvements. The number of applicants last year was so great that beds were placed in the men's dining-room and meals served in the gallery, which is wide and spacious, on the top floor; here are a number of small apartments originally planned for the better class of inmates. Practically at present they are useless, and it is proposed to convert them into sleeping wards. The number of inmates varies; last year it was great owing to depression of trade. At the end of March last about 650. The diet scale is liberal and ample, consisting largely of rice, plantains, cassava, and yams.

The majority of the inmates are negroes and coloured people of various races, a few whites, among them an old Irishwoman, who had been an inmate over 20 years, and a number of East Indians or coolies. It is a troublesome task to deal with coolie women, as they are dirty in their habits. The authorities are anxious to do their best, and inmates are kindly treated as far as circumstances allow; indeed coloured people are usually happy if allowed to lie and bask in the sun with food and nothing to do.

There is a resident dispenser in this block and good medical attendance. A new matron was appointed last May, but I do not know if any reforms have taken place; certainly there is a splendid field for better nursing, but any scheme to be successful means extra expenses.

